# Machine learning-based treatment outcome prediction in head and neck cancer using integrated noninvasive diagnostics

**DOI:** 10.1007/s11548-025-03539-2

**Published:** 2025-12-08

**Authors:** Melda Yeghaian, Stefano Trebeschi, Marina Herrero-Huertas, Francisco Javier Mendoza Ferradás, Paula Bos, Maarten J. A. van Alphen, Marcel A. J. van Gerven, Regina G. H. Beets-Tan, Zuhir Bodalal, Lilly-Ann van der Velden

**Affiliations:** 1 Department of Radiology, The Netherlands Cancer Institute, Amsterdam, The Netherlands; 2 GROW Research Institute for Oncology and Reproduction, Maastricht University, Maastricht, Limburg The Netherlands; 3 Department of Radiology, Hospital Fundacion Jimenez-Diaz, Madrid, Spain; 4 Department of Vascular and Interventional Radiology, Hospital Universitario de Navarra, Pamplona, Navarra Spain; 5 Department of Radiotherapy, The Netherlands Cancer Institute, Amsterdam, The Netherlands; 6 Department of Head and Neck Oncology and Surgery, The Netherlands Cancer Institute, Amsterdam, The Netherlands; 7 Department of Machine Learning and Neural Computing, Donders Institute for Brain, Cognition and Behaviour, Radboud University, Nijmegen, Gelderland The Netherlands; 8 Faculty of Health Science, University of Southern Denmark, Odense, Denmark; 9 The Netherlands Cancer Institute, Amsterdam, The Netherlands

**Keywords:** Head and neck cancer, Machine learning, Postsurgical outcomes, Integrative diagnostics, Survival prediction, Feeding tube dependence prediction

## Abstract

**Purpose:**

Accurate prediction of treatment outcomes is crucial for personalized treatment in head and neck squamous cell carcinoma (HNSCC). Beyond one-year survival, assessing long-term enteral nutrition dependence is essential for optimizing patient counseling and resource allocation. This preliminary study aimed to predict one-year survival and feeding tube dependence in surgically treated HNSCC patients using classical machine learning.

**Methods:**

This proof-of-principle retrospective study included 558 surgically treated HNSCC patients. Baseline clinical data, routine blood markers, and MRI-based radiomic features were collected before treatment. Additional postsurgical treatments within one year were also recorded. Random forest classifiers were trained to predict one-year survival and feeding tube dependence. Model explainability was assessed using Shapley Additive exPlanation (SHAP) values.

**Results:**

Using tenfold stratified cross-validation, clinical data showed the highest predictive performance for survival (AUC = 0.75 ± 0.10; *p* < 0.001). Blood (AUC = 0.67 ± 0.17; *p* = 0.001) and imaging (AUC = 0.68 ± 0.16; *p* = 0.26) showed moderate performance, and multimodal integration did not improve predictions (AUC = 0.68 ± 0.16; *p* = 0.38). For feeding tube dependence, all modalities had low predictive power (AUC ≤ 0.66; *p *> 0.05). However, postsurgical treatment information outperformed all other modalities (AUC = 0.67 ± 0.07; *p *= 0.002), but had the lowest predictive value for survival (AUC = 0.57 ± 0.11; *p* = 0.08).

**Conclusion:**

Clinical data appeared to be the strongest predictor of one-year survival in surgically treated HNSCC, although overall predictive performance was moderate. Postsurgical treatment information played a key role in predicting tube feeding dependence. While multimodal integration did not enhance overall model performance, it showed modest gains for weaker individual modalities, suggesting potential complementarity that warrants further investigation.

**Supplementary Information:**

The online version contains supplementary material available at 10.1007/s11548-025-03539-2.

## Introduction

Head and neck squamous cell carcinoma (HNSCC) is the seventh most common cancer worldwide, accounting for approximately 4.5% of all cancer diagnoses and 4.6% of cancer-related deaths globally [1]. It originates from the mucosal linings of the oral cavity, oropharynx, larynx, or hypopharynx [2, 3]. Common risk factors include tobacco use, alcohol consumption, human papillomavirus (HPV) infection, and, in some regions, Epstein–Barr virus [1, 3]. Treatment options include surgery, radiotherapy, chemotherapy, or multimodal regimens [3, 4].

Several clinicopathological factors—most notably the TNM classification and HPV status—have proven instrumental in estimating HNSCC prognosis [5, 6]. Additionally, liquid and imaging-based biomarkers have gained traction due to their noninvasive nature and their potential to provide unique biological insights [5]. While traditional prognostic models, such as TNM staging and HPV status, provide valuable insights, they often fail to capture the full heterogeneity of the disease. Emerging biomarkers from routinely used medical data sources in daily clinical practice, such as radiological imaging and blood tests, present promising opportunities to enhance prediction accuracy.

In addition to prognosis, tube feeding dependence is one functional outcome that can profoundly affect quality of life in HNSCC. Patients often require enteral nutrition support when weight loss, malnutrition, dysphagia, or other complications impair oral intake, ensuring adequate nutritional intake [7–11]. Being able to predict which surgically treated patients will need a feeding tube one year postoperatively would allow clinicians to optimize care pathways, tailor rehabilitation, and allocate resources more effectively.

Accurately predicting HNSCC treatment outcomes, particularly survival and feeding tube dependence, remains challenging due to the complex interplay of clinical, biological, and treatment-related factors. Recently, artificial intelligence (AI) methods have begun to harness high-dimensional, multimodal data routinely collected in clinical practice, such as advanced imaging techniques and electronic health records [12]. These integrative approaches may help enhance the accuracy of survival and functional outcome predictions and could eventually contribute to improved patient stratification and supportive care once validated. In this preliminary study, we explore the feasibility of such integration by combining clinical, blood, and MRI-based radiomic data, along with information on postsurgical treatments, to develop machine learning models for predicting one-year survival and feeding tube dependence in HNSCC patients.

## Materials and methods

### Study cohort and ethical approval

We retrospectively collected data from all patients with HNSCC treated at the Netherlands Cancer Institute between January 2008 and July 2018. Institutional review board approval was obtained from the Netherlands Cancer Institute (IRBd20-082), where the need for project-specific informed consent was waived. We included patients who underwent surgery as their initial treatment approach. Patients who were lost to follow-up, had multiple simultaneous tumors, or had a prior head and neck tumor were excluded.

For each included patient, we gathered baseline pretreatment data, including clinical parameters, laboratory blood test results, and magnetic resonance (MR) imaging. Specifically, T1-weighted (T1W) MR scans with contrast enhancement were retrieved. We also recorded survival status and dates of death, as well as information regarding the placement of feeding tubes during and after treatment. Additionally, we documented whether patients received any other treatments within the prediction window. The study’s primary outcomes were formulated as two binary classification tasks: (1) predicting one-year overall survival and (2) determining the need for a feeding tube one year after surgery.

### Data preprocessing and machine learning analysis

All collected data were preprocessed into numerical features. Two radiologists (MHH and FG) generated volumetric segmentations of the primary head and neck tumors on MR imaging using 3D Slicer [13]. Radiomic features were extracted from the contrast-enhanced T1W MR scans using these segmentations. Missing values within the clinical and blood data were handled using multivariate iterative imputation from scikit-learn [14]. This approach models each variable with missing values as a regression problem using the other available variables as predictors. The procedure cycles through all incomplete variables in a round-robin fashion until convergence, producing plausible estimates that account for multivariate relationships. The resulting data were standardized to have a mean of zero and a unit variance. Further details of the extracted features can be found in Supplementary Materials 1.

To assess whether these data could predict one-year treatment outcomes, namely survival and feeding tube placement, we trained 10 random forest classifiers for each outcome using tenfold stratified cross-validation (CV). Hyperparameter tuning for a subset of random forest parameters (Supplementary Materials 2) was performed using the halving randomized search strategy across all 10 CV folds during training [14]. Class imbalance within each training fold was addressed through combined over- and under-sampling using SMOTE and Tomek links [15]. Shapley Additive exPlanation (SHAP) values were used to explain the decisions of the random forest classifier based on the validation sets [16]. Analyses were conducted using Python v3.10.13 and the following packages: scikit-learn v1.3.0 [14], NumPy v1.26.0, Pandas v2.0.3, PyRadiomics v3.0.1 [17], Imbalanced-learn v0.12.2 [18], and SHAP v0.42.1 [16].

### Evaluation approach

The performance of each random forest model was assessed using the average area under the receiver operating characteristic curve (ROC-AUC), the area under the precision–recall curve (PR-AUC), and the F1-score at the median performance across the 10 CV folds. We also report the standard deviations for these metrics across the 10 CV folds. Statistical significance was determined using the Mann–Whitney U test in each CV fold, and p-values were then combined using Fisher’s method; *p* < 0.05 was considered statistically significant.

We further examined whether additional treatments (subsequent or adjuvant) received within the first year postoperatively influenced model performance. Each data modality (clinical, laboratory, and imaging) was analyzed both independently and in combination, with the presence of additional treatments included as a binary feature. This approach enabled us to assess the additive value of each modality and the potential confounding effects of additional interventions.

In our main experiments, we used 107 radiomic features extracted from the original image. Given the high dimensionality of the full radiomic feature set (*n* = 1874), additional evaluations were conducted using all features, different subsets of the top-selected features (*k* = 40 and 100), and a refined set excluding correlated features (*n* = 84, median), as detailed in Supplementary Table 1.

## Results

### Patient characteristics

A total of 1761 HNSCC patients were initially screened between January 2008 and July 2018. From these, we included only 728 patients who underwent surgery as their first treatment. We excluded 19 patients who were lost to follow-up, 53 with multiple simultaneous tumors, and 98 who had previous head and neck tumors, resulting in a final cohort of 558 patients. Among these, 243 received additional treatments (*e.g*., adjuvant or subsequent therapy) within the first postsurgical year. A total of 354 patients (63%) were male with a median age of 64 (interquartile range: 56–73).

While all 558 patients had at least 25% of the included clinical parameters and routine blood markers, only 195 had T1W contrast-enhanced MR imaging available. We included 34 clinical parameters, 18 blood markers, and 107 radiomic features in our study. A total of 478 patients (86%) in the included cohort survived within one year from surgery, whereas only 52 patients (9%) required a feeding tube after one year. Table 1 summarizes the baseline patient characteristics and the data modalities used.

**Table 1:** Baseline demographic and clinical characteristics of the complete 558 HNSCC patient cohort, along with the availability of data modalities for model development

Characteristics	N patients (%)
Age	Median (interquartile range)
Years	64 (56–73)
Sex	
Male	354 (63%)
Female	204 (37%)
Diagnosis anatomical region	
Oral cavity	341 (61%)
Larynx	134 (24%)
Oropharynx	64 (11.5%)
Hypopharynx	14 (2.5%)
Salivary gland	5 (1%)
Treatment	
Surgery	558 (100%)
Additional treatments after the initial surgery in one year	243 (44%)
Outcomes at 1 year	
Survival	478 (86%)
Feeding tube requirement	52 (9%)
Data modalities	
Clinical data	558 (100%)
Routine blood markers	558 (100%)
Imaging	195 (35%)
All modalities	195 (35%)
Number of features	N features
Clinical data	34
Routine blood markers	18
Imaging	107
All modalities	159
Missing values	Maximum % in a feature
Clinical data	62%
Routine blood markers	73.5%

### Predicting treatment outcomes

We investigated the predictive value of each pretreatment data modality—clinical data, blood-based markers, and imaging features—both independently and in combination, for two binary classification tasks: (1) one-year survival post-surgery and (2) one-year dependence on tube feeding. Model performance was evaluated using the area under the receiver operating characteristic curve (AUC), the area under the precision–recall curve (PR-AUC), and the F1-score.

### Predicting one-year overall survival

Without including postsurgical treatment information, clinical data alone demonstrated the highest predictive power for one-year survival (AUC: 0.75 ± 0.10, PR-AUC: 0.94 ± 0.03, F1-score: 0.64 ± 0.06; *p* < 0.001). Blood-based parameters showed moderate predictive ability (AUC: 0.67 ± 0.09, PR-AUC: 0.93 ± 0.02, F1-score: 0.68 ± 0.05; *p* = 0.001). In contrast, imaging features alone yielded a moderate AUC of 0.67 ± 0.17 but did not reach statistical significance (*p* = 0.26). Notably, combining all three modalities did not improve performance beyond that achieved by clinical data alone (AUC: 0.68 ± 0.16; *p* = 0.38).

When information regarding additional postsurgical treatments was incorporated into the model as a binary feature, the predictive performance of the prognostic model did not meaningfully change. Clinical data continued to yield the best survival predictions, with a slight increase in AUC (0.76 ± 0.07) and PR-AUC (0.95 ± 0.02). Blood data performance remained relatively stable, showing only a minor decrease in AUC (0.66 ± 0.09, PR-AUC: 0.92 ± 0.03; *p* = 0.002). Imaging data again provided modest predictive capability (AUC: 0.64 ± 0.16, PR-AUC: 0.92 ± 0.05, F1-score: 0.65 ± 0.08; *p* = 0.45). Importantly, adding imaging and blood variables to clinical data did not confer additional improvements (AUC: 0.66 ± 0.12, PR-AUC: 0.92 ± 0.04; *p* = 0.62).

Predicting one-year survival using information on whether the patient received additional postsurgical treatment data alone yielded the lowest performance (AUC: 0.57 ± 0.11, PR-AUC: 0.91 ± 0.03, F1-score: 0.13 ± 0.27; *p* = 0.08), indicating that merely knowing whether patients received additional therapies was insufficient for robust survival prediction (Table 1).

### Predicting dependence on tube feeding

Predicting feeding tube requirements one year after surgery was more challenging than predicting survival (Table 2). Before incorporating additional postsurgical treatment information, clinical data offered the highest AUC (0.66 ± 0.07) but had a low PR-AUC (0.23 ± 0.12) and an F1-score of 0.22 ± 0.06, without reaching statistical significance. Blood (AUC: 0.64 ± 0.08) and imaging (0.60 ± 0.24) had similar or lower performance, though imaging features had a slightly higher PR-AUC (0.28 ± 0.16). Combining all modalities did not markedly improve performance (AUC: 0.65 ± 0.19, *p* = 0.48).

**Table 2:** Performance of random forest models for predicting one-year survival using individual (clinical, blood, and imaging) and combined modalities across 10 cross-validation folds (with and without the inclusion of additional postsurgical treatment data)

Additional treatments	Modality	N (patients)	AUC	PR-AUC	F1-score	*p*-value
Not included	Clinical	558	**0.75 ± 0.10**	**0.94 ± 0.03**	0.64 ± 0.06	< 0.001
	Blood	558	0.67 ± 0.09	0.93 ± 0.02	**0.68 ± 0.05**	0.001
	Imaging	195	0.67 ± 0.17	0.92 ± 0.06	**0.68 ± 0.06**	0.26
	Combined	195	0.68 ± 0.16	0.92 ± 0.06	**0.68 ± 0.05**	0.38
Included	Additional treatments	558	0.57 ± 0.11	0.91 ± 0.03	0.13 ± 0.27	0.08
Included	Clinical	558	**0.76** ± **0.07**	**0.95 ± 0.02**	0.66 ± 0.07	< 0.001
	Blood	558	0.66 ± 0.09	0.92 ± 0.03	**0.68 ± 0.06**	0.002
	Imaging	195	0.64 ± 0.16	0.92 ± 0.05	0.65 ± 0.08	0.45
	Combined	195	0.66 ± 0.12	0.92 ± 0.04	0.67 ± 0.06	0.62

**Fig. 1:**
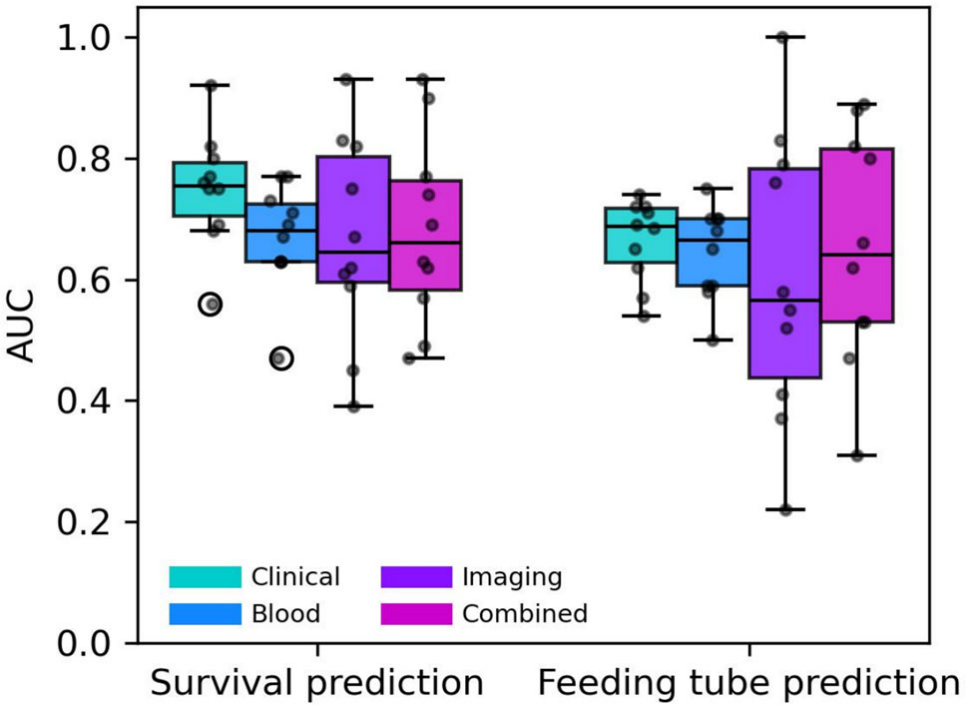
Predictive performance of models trained on individual and combined data modalities. Boxplots of the area under the receiver operating characteristic curve (AUC) across 10 cross-validation folds for both (left) one-year survival and (right) one-year feeding tube dependence predictions. Predictive models were evaluated using (turquoise) clinical data, (blue) blood markers, (purple) imaging features, and (magenta) all modalities combined

After including details about additional postsurgical treatments, performance for feeding tube prediction improved, particularly for blood data (AUC: 0.70 ± 0.12, PR-AUC: 0.22 ± 0.09; *p* = 0.001) and clinical data (AUC: 0.69 ± 0.07, PR-AUC: 0.22 ± 0.08; *p* = 0.01). Imaging performance remained largely unchanged (AUC: 0.61 ± 0.20, *p* = 0.77), while the combined model showed a slight decrease (AUC: 0.63 ± 0.22, *p* = 0.48). Interestingly, models using only the binary “presence of additional postsurgical treatment” variable achieved an AUC of 0.67 ± 0.07 (PR-AUC: 0.47 ± 0.08; *p* = 0.002), suggesting that postoperative treatment patterns may contain meaningful information relevant to nutritional support needs (Table 3 and Fig. 1).

**Table 3:** Performance of random forest models for predicting one-year feeding tube dependence using individual (clinical, blood, and imaging) and combined modalities across 10 cross-validation folds (with and without the inclusion of additional postsurgical treatment data)

Other treatments	Modality	N (patients)	AUC	PR-AUC	F1-score	p-value
Not included	Clinical	558	**0.66 ± 0.07**	0.23 ± 0.12	0.22 ± 0.06	0.07
	Blood	558	0.64 ± 0.08	0.15 ± 0.04	0.22 ± 0.06	0.17
	Imaging	195	0.60 ± 0.24	**0.28 ± 0.16**	**0.24 ± 0.30**	0.57
	Combined	195	0.65 ± 0.19	0.22 ± 0.20	0.23 ± 0.13	0.48
Included	Additional treatments	558	0.67 ± 0.07	0.47 ± 0.08	0.21 ± 0.12	0.002
Included	Clinical	558	0.69 ± 0.07	**0.22 ± 0.08**	0.23 ± 0.04	0.01
	Blood	558	**0.70 ± 0.12**	**0.22 ± 0.09**	**0.24 ± 0.09**	0.001
	Imaging	195	0.61 ± 0.20	**0.22** ± **0.41**	0.20 ± 0.08	0.77
	Combined	195	0.63 ± 0.22	0.21 ± 0.19	0.17 ± 0.13	0.48

### Explainability of the classifiers

We used SHAP values to identify the most influential features in predicting both survival and feeding tube dependence.

**Fig. 2:**
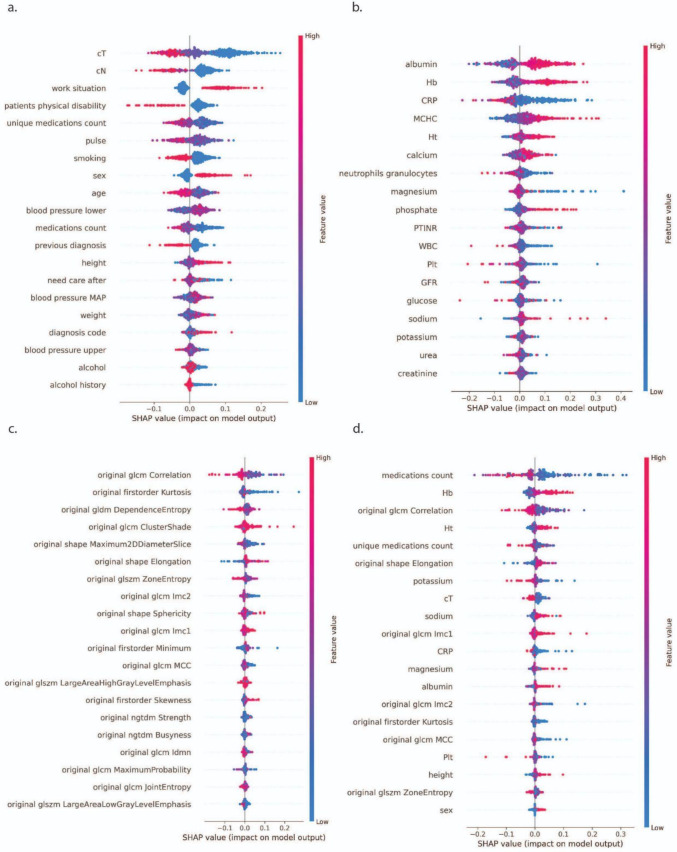
Explainability of survival models. SHAP summary plots showing the most influential features for predicting one-year survival when using **a** clinical data, **b** blood markers, **c** imaging features, and **d** all modalities combined. These analyses do not include additional postsurgical treatment data

With respect to predicting survival, the interpretation of most influential features is as follows. In the clinical model, T stage and N stage consistently emerged as the strongest survival predictors (Table 4). T stage corresponds to the tumor size and extent, while N refers to the degree of lymph node involvement. Functional or lifestyle factors (*e.g*., work situation, physical disability) also remained relevant. After including postsurgical treatment information, heart rate (pulse) appeared among the top five features, indicating potential physiological influences. For blood biomarkers, albumin, hemoglobin (Hb), C-reactive protein (CRP), and mean corpuscular hemoglobin concentration (MCHC) were key predictors, highlighting the impact of systemic inflammation and nutritional status. The binary “additional treatments” feature also emerged as an important variable when included. Gray-level co-occurrence matrix (GLCM) correlation and first-order kurtosis consistently ranked among the top radiomic predictors. Shape-based metrics, such as maximum 2D diameter (slice), were also important before and after including postsurgical treatment information. When all modalities were combined, clinical and blood-based features (*e.g*., medication count, unique medications count, and Hb) dominated the combined model’s top rankings. Among the imaging features, GLCM correlation and gray-level dependence matrix (GLDM) dependence entropy were the most influential, hinting at the importance of tumor texture heterogeneity for survival prediction. Figure 2 illustrates the ranked predictors for survival in SHAP summary plots.

**Table 4:** Top five SHAP-ranked features (in order of importance) driving one-year survival prediction for each data modality (clinical, blood, imaging, and combined), reported with and without the presence of additional postsurgical treatments as a binary feature

Other treatments	Modality	Predictive feature 1	Predictive feature 2	Predictive feature 3	Predictive feature 4	Predictive feature 5
Not included	Clinical	T stage	N stage	Work situation	Physical disability	Unique medications count
	Blood	Albumin	Hemoglobin	CRP	MCHC	Hematocrit
	Imaging	Correlation (GLCM)	Kurtosis (first order)	Dependence entropy (GLDM)	Cluster shade (GLCM)	Maximum 2D diameter slice (shape)
	Combined	Medications count	Hemoglobin	Correlation (GLCM)	Hematocrit	Unique medications count
Included	Clinical	T stage	N stage	Patients physical disability	Work situation	Heart rate
	Blood	Albumin	Hemoglobin	CRP	MCHC	Additional treatments
	Imaging	Correlation (GLCM)	Zone entropy (GLSZM)	Kurtosis (first order)	Elongation (shape)	Maximum 2D diameter slice (shape)
	Combined	Medications count	Hemoglobin	Unique medications count	Correlation (GLCM)	Dependence entropy (GLDM)

**Fig. 3:**
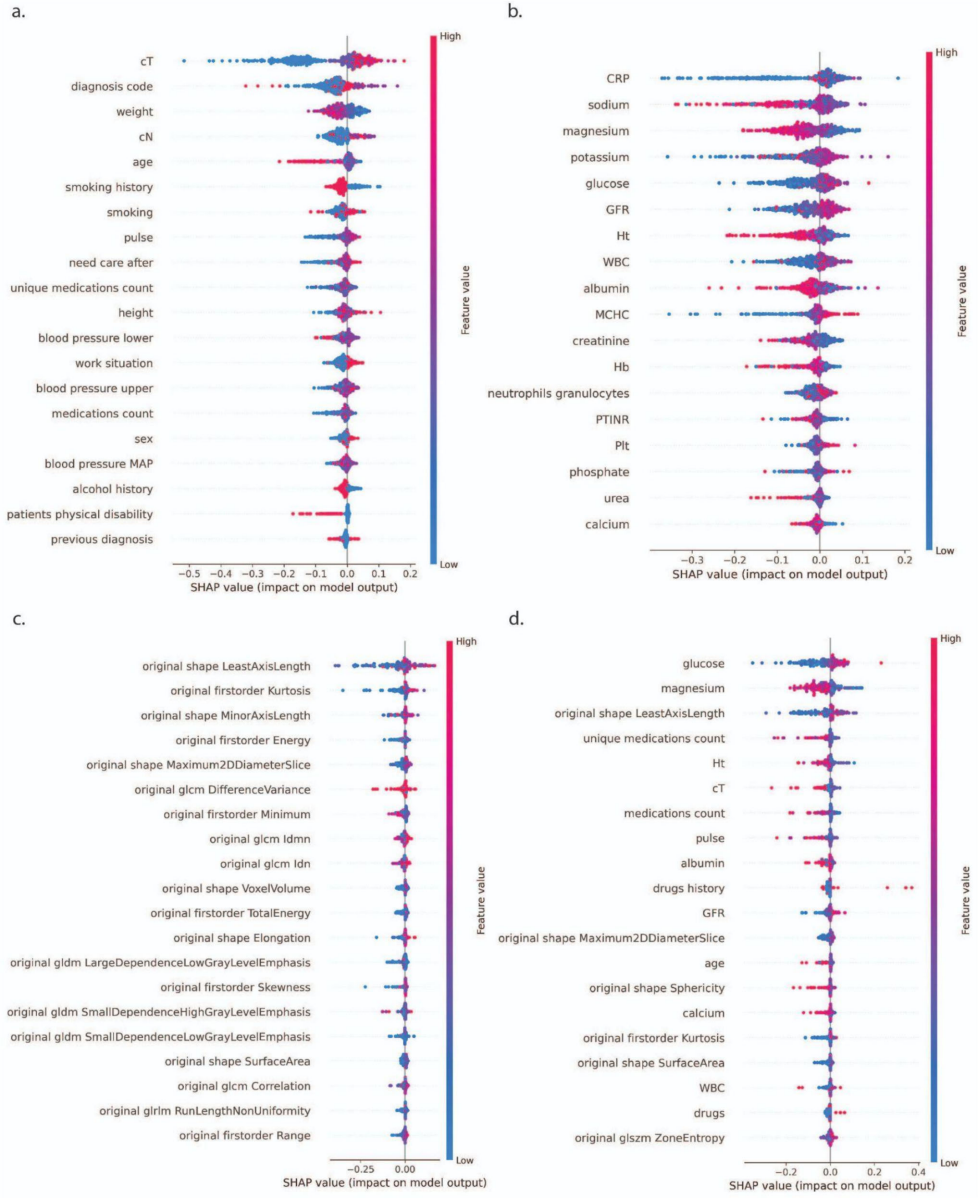
Explainability of feeding tube prediction models. SHAP summary plots illustrating key predictors of one-year feeding tube dependence using **a** clinical data, **b** blood markers, **c** imaging features, and **d** all modalities combined, without incorporating additional postsurgical treatment information

With respect to predicting dependence on tube feeding, the interpretation of the most influential features is as follows. Before accounting for postsurgical treatment details, clinical T stage, diagnosis type, weight, and age had the highest impact (Table 5 and Fig. 3). For blood-based predictors, CRP, sodium, magnesium, potassium, and glucose repeatedly appeared as top predictors, reinforcing the role of systemic inflammation, electrolyte balance, and metabolic status in prolonged feeding tube dependence. In the imaging model, shape-based features were the most predictive. Least axis length, minor axis length, and maximum 2D diameter slice were among the strongest predictors, suggesting that anatomical structures contributed to feeding tube dependency. When all modalities were combined, metabolic markers (*e.g*., glucose, magnesium) and shape-based features (*e.g*., least axis length) were important before additional treatment information was taken into account. Once postsurgical treatment information was added to all individual and combined modalities, the “additional treatments” feature became the leading predictor, suggesting that subsequent interventions significantly influenced whether patients developed feeding tube dependence.

**Table 5:** Top five SHAP-ranked features (in order of importance) influencing one-year feeding tube dependence prediction under each data modality (clinical, blood, imaging, and combined), both with and without incorporating additional postsurgical treatments as a potential confounder

Other treatments	Modality	Predictive feature 1	Predictive feature 2	Predictive feature 3	Predictive feature 4	Predictive feature 5
Not included	Clinical	T stage	Diagnosis	Weight	N stage	Age
	Blood	CRP	Sodium	Magnesium	Potassium	Glucose
	Imaging	Least axis length (shape)	Kurtosis (first order)	Minor axis length (shape)	Energy (first order)	Maximum 2D diameter slice (shape)
	Combined	Glucose	Magnesium	Least axis length (shape)	Unique medications count	Hematocrit
Included	Clinical	Additional treatments	T stage	Diagnosis	Weight	N stage
	Blood	Additional treatments	CRP	Sodium	Magnesium	Potassium
	Imaging	Additional treatments	Least axis length (shape)	Kurtosis (first order)	Minor axis length (shape)	Elongation (shape)
	Combined	Additional treatments	Magnesium	Glucose	Least axis length (shape)	Albumin

## Discussion

Head and neck squamous cell carcinoma poses persistent challenges regarding morbidity, mortality, and patient quality of life. Surgical resection is a cornerstone of treatment for many patients, yet predicting longer-term outcomes, especially survival and feeding tube dependence, remains difficult. Patient stratification is crucial for optimizing clinical decision-making, guiding supportive care, and informing resource allocation. In this preliminary study, we applied machine learning models to combine routinely collected clinical data, blood markers, and T1W MR imaging radiomics to examine their potential for predicting one-year survival and feeding tube dependence in surgically treated HNSCC patients. We further investigated whether including the presence of additional postsurgical treatments (as a binary feature) could provide any potential added value to the analyzed outcomes, given the availability of this information in our retrospective dataset.

The prognostic performance of one-year survival prediction models varied across modalities. Clinical data alone proved to be the strongest predictor of survival, consistently achieving the highest AUCs and statistically significant performance. Tumor T stage and N stage were ranked the highest via SHAP analysis. Several studies demonstrated an association between TNM staging and survival outcomes, with earlier stages correlating with longer survival times [19]. Our study also showed that lower T and N stages are more likely to predict patient survival at one year. Blood-based laboratory markers exhibited moderate prognostic capacity. SHAP analysis suggested that higher levels of albumin, hemoglobin (Hb), and lower levels of CRP were linked to patient survival. Similarly, Valero et al. showed that lower albumin levels were associated with poorer survival outcomes in patients with oral cavity squamous cell carcinoma [20]. Although MRI-based radiomics displayed only modest performance and did not appreciably enhance survival predictions beyond clinical data, certain texture features (*e.g*., GLCM correlation) were identified as important potential predictors. This observation echoes earlier radiomics research in HNSCC, which highlighted various texture features (among which GLCM correlation) as relevant to survival prediction [21].

In contrast to survival, predicting one-year feeding tube dependence proved more challenging across all modalities, likely reflecting complex and multifactorial determinants of long-term nutritional needs in surgically treated patients. Imbalanced outcome data further complicated model performance. This may also be because many head and neck cancer patients require feeding tubes during other treatment types, such as radiotherapy or chemo–radiotherapy, due to treatment-related toxicity [22, 23]. This hypothesis was promptly assessed by incorporating the presence of additional postsurgical treatments as a feature alongside the existing features within each modality and evaluating its individual ability to predict feeding tube dependence at one year. Our results (Table 3) demonstrated that this single feature alone outperformed individual modalities that did not include this information (feature). It also improved the performance of all individual modalities (in combination with this feature) for predicting one-year feeding tube dependence and was ranked the first predictor by SHAP analysis. Capturing the specific nature and timing of different treatments, rather than using a single binary flag, may yield even deeper insights into the risk of feeding tube dependence in future studies.

While combining all three modalities (clinical, blood, and imaging) provided a more comprehensive profile of patient health, it did not outperform the best-performing individual modality for either survival or feeding tube dependence prediction. Despite these findings, we observed that weaker individual modalities (*e.g*., imaging alone) could see incremental improvements when combined with the other stronger modalities (*e.g*., clinical data). This may be due to differences in data representation, as radiomic features capture detailed spatial information, whereas certain clinical features, such as TNM staging, are represented as more general categorical classifications. However, an unexpected “dilution” effect arose when all modalities were combined, suggesting that non-informative or redundant features may have overshadowed the most meaningful potential predictors in a relatively small dataset. This phenomenon, where irrelevant or redundant input features impede classifier performance, is known as the curse of dimensionality in machine learning [24]. Therefore, it deserves more attention when considering the integration of multimodal health data in general. This dilution effect may, in part, also stem from differences in data representation, as the model had to integrate categorical clinical features with continuous radiomic features, potentially affecting the balance and contribution of each modality. We addressed this redundancy in the extracted radiomic features by focusing solely on non-filtered (original) image features. Conducting additional experiments applying classical feature selection methods instead, such as removing correlated features or selecting the top *k* = 100 features from all radiomic feature types (*N* = 1874, Supplementary Materials 1), also failed to improve integrative performance (Supplementary Table 1). The lack of significant improvement in combined modalities could also reflect an underlying biological limitation: Certain predictive signals from different modalities might not be additive or synergistic. For example, imaging-derived features and blood biomarkers may capture overlapping physiological processes, diminishing the value of combining them without targeted selection of complementary features.

Previous studies have integrated multiple data modalities to predict overall survival in head and neck cancer. For instance, Mes et al. [25] and Bos et al. [26] combined clinical data with MRI-based radiomics to predict overall survival in patients with HNSCC, demonstrating improved performance when both modalities were used together. In contrast, our study also incorporated blood markers alongside clinical data and radiomics, yet the combination did not improve predictive performance. Differences in the patient cohort may influence this outcome, as our study focused on surgically treated patients, whereas other studies have focused on patients receiving chemotherapy, radiotherapy, or non-surgical treatments. Another integration study in HNSCC patients treated with chemo–radiotherapy also showed that combining radiomics derived from CT and FDG-PET pretreatment scans did not improve performance in predicting locoregional recurrence [27].

Although we highlighted results for survival and feeding tube dependence prediction, this study has limitations that merit consideration. First, this is a single-institution, retrospective study, without external validation, which may limit generalizability. To partially address this limitation, we used cross-validation strategies to provide an internal assessment of model performance. Second, incomplete MRI data restricted the sample size for radiomics-based analyses, potentially introducing bias. Even after feature selection, the number of radiomic features remained relatively high compared with the sample size, which may reduce the robustness of our findings. Additionally, missing values within the clinical and blood datasets required multivariate iterative imputation, which, although leveraging relationships among variables, may introduce bias because imputed values are model-based estimates rather than true measurements. Such challenges are common in multimodal AI research, where the availability of at least one modality or complete data can limit the overall study size and influence model outcomes. Third, despite employing SMOTE and Tomek links, the class imbalance in feeding tube dependence likely reduced model performance. Additionally, the scope of “additional treatments” was captured as a binary feature (present/absent) rather than specifying treatment type (*e.g*., adjuvant radiotherapy vs. salvage surgery). Such granularity could offer further insights into how different postsurgical regimens affect nutritional support needs. Looking forward, further research should focus on expanding data collection to include larger multicenter cohorts that capture a wider range of treatment approaches. Incorporating more granular postsurgical treatment details (*e.g*., radiotherapy fields, chemotherapy regimens) and exploring longitudinal features (*e.g*., repeated blood marker measurements or serial imaging) may also provide more refined predictions of functional outcomes, such as feeding tube dependence.

Notwithstanding these limitations, these preliminary findings suggest that integrating clinical, blood-based, and imaging data, along with insights into subsequent treatments, may provide complementary insights into patient outcomes in HNSCC. In practice, combining multiple data modalities may not always improve the performance of all classical AI models. Future research with larger cohorts, more complete and complementary data, refined predictive features, exploration of advanced AI methods, and external validation sets will be needed to develop more robust and generalizable models for survival and functional outcome prediction, prior to any consideration of clinical translation.

## Supplementary Information

Below is the link to the electronic supplementary material.

Supplementary file1 (DOCX 230 KB)
