# Supplementary material for: Machine learning-based treatment outcome prediction in head and neck cancer using integrated noninvasive diagnostics
Source: Int J Comput Assist Radiol Surg. 2025 Dec 8;21(3):483–93. doi: 10.1007/s11548-025-03539-2 (PMC13035643; doi:10.1007/s11548-025-03539-2)

Machine Learning-Based Treatment Outcome Prediction in Head and Neck Cancer Using Integrated Noninvasive Diagnostics

Melda Yeghaian^1, 2^, Stefano Trebeschi^1,2^, Marina Herrero-Huertas^3^, Francisco Javier Mendoza Ferradás^4^, Paula Bos^5^, Maarten J A van Alphen^6^, Marcel A J van Gerven^7^, Regina G H Beets-Tan^1, 2, 8^, Zuhir Bodalal^1, 2,*^, Lilly-Ann van der Velden ^6, *^

^1^ Departments of Radiology,^5^ Radiotherapy, and ^6^ Head and Neck Oncology and Surgery, The Netherlands Cancer Institute, Amsterdam, The Netherlands.

^2^ GROW Research Institute for Oncology & Reproduction, Maastricht University, Maastricht, Limburg, The Netherlands.

^3^ Department of Radiology, Hospital Fundacion Jimenez-Diaz, Madrid, Spain

^4^ Department of Vascular and Interventional Radiology, Hospital Universitario de Navarra, Pamplona, Navarra, Spain

^7^ Department of Machine Learning and Neural Computing, Donders Institute for Brain, Cognition and Behaviour, Radboud University, Nijmegen, Gelderland, The Netherlands.

^8^ Faculty of Health Science, University of Southern Denmark, Denmark.

* These authors contributed equally and are considered joint last authors

# * **Corresponding Author** Zuhir Bodalal, GROW School for Oncology and Developmental Biology, Maastricht University, Maastricht, 6229 ER, Limburg, The Netherlands. E-mail: z.elkarghali@maastrichtuniversity.nl.

#

# **Supplementary materials**

## **Supplementary Materials 1.** *Comprehensive list and definitions of the clinical features, routine blood markers, and extracted radiomic features.*

For each patient, a total of 34 clinical features were extracted, namely: age, sex, height, weight, HPV status, p16 status, heart rate (pulse), need for walking aid, whether the patient received help at home, upper bound of blood pressure, lower bound of blood pressure, mean arterial pressure (MAP), the number of prescribed medications, the number of unique prescribed medications, diagnosis, whether the patient needed help with mobility, whether the patient is working, whether the patient lives independently, whether the patient had any previous diagnosis of non-head and neck cancer, presence of any comorbidities, T stage (cT), N stage (cN), M stage (cM), current smoking status, previous smoking status, current drug use, previous drug use, current alcohol consumption, previous alcohol consumption, unintentional weight loss, weight loss in the last 6 months, whether patient thinks they will need care after, whether the patient has any other disorders, presence of any physical disability.

We also included measurements of 18 routine blood markers, including, albumin, calcium, C-reactive protein (CRP), phosphate, GFR, glucose, hemoglobin (Hb), hematocrit (Ht), potassium, creatinine, MCHC, magnesium, sodium, neutrophil granulocytes, thrombocytes (Plt), prothrombin time with international normalized ratio (PT/INR), urea, leukocytes (WBC).

Radiomic feature extraction was performed using multiple image types and filters, including the original image, Laplacian of Gaussian (LoG) with sigma values of 2.0, 4.0, and 5.0, and wavelet-transformed images. Additionally, image transformations such as Square, SquareRoot, Logarithm, Exponential, and Gradient were applied. A 3D Local Binary Pattern (LBP3D) filter was also utilized. These features encompassed all major categories, including first-order statistical features, shape- and size-based features, as well as second-order textural features derived from matrices like the Gray Level Co-occurrence Matrix (GLCM), Gray Level Run Length Matrix (GLRLM), Neighboring Gray Tone Difference Matrix (NGTDM), and Gray Level Dependence Matrix (GLDM). These resulted in the extraction of a total of 1874 radiomic features. Detailed explanations of these features can be found in the PyRadiomics documentation and the work by Griethuysen et al. [[17]](https://paperpile.com/c/qW9B6d/G2j7). Given the high dimensionality of the extracted radiomic features (n=1874 features), only the features extracted from the original image (n=107) were utilized within the main manuscript.

## **Supplementary Materials 2.** *Comprehensive list of the hyperparameters tuned during the training of the random forest models, along with their associated values.*

We tuned the following random forest hyperparameters: max_depth (None, 10, 20, 30), min_samples_split (2, 5, 10), min_samples_leaf (1, 2, 4), max_features (sqrt, log2, None), and class_weight (balanced, balanced_subsample). Cross-validation using halving randomized search on hyperparameters was implemented using up to 200 n_estimators (trees). Default values were used for the remaining hyperparameters.

#

**Supplementary Table 1**. One-year treatment outcome prediction for n=195 head and neck cancer patients using different variants of radiomic features and combined data modalities across 10 cross-validation folds (without incorporating any additional treatment information). * indicates the median number of features

| Prediction Task | Modality | Features description | N (features) | AUC | PR-AUC | F1-score | p-value |
| --- | --- | --- | --- | --- | --- | --- | --- |
| Survival | Imaging | all | 1874 | 0.57±0.24 | 0.87±0.10 | 0.64±0.10 | 0.20 |
|  |  | original | 107 | **0.67±0.17** | **0.92±0.06** | **0.68±0.06** | 0.26 |
|  |  | selected k best | 40 | 0.61±0.13 | 0.90±0.05 | 0.67±0.07 | 0.81 |
|  | Combined | all | 1926 | **0.69±0.17** | **0.92±0.07** | 0.67±0.09 | 0.31 |
|  |  | imaging (original) | 159 | 0.68±0.16 | **0.92±0.06** | **0.68±0.05** | 0.38 |
|  |  | selected k best | 100 | 0.63±0.15 | 0.91±0.06 | 0.68±0.07 | 0.62 |
|  |  | excluding correlated features | 84* | 0.67±0.14 | 0.92±0.07 | 0.67±0.10 | 0.38 |
| Feeding tube | Imaging | all | 1874 | **0.66±0.11** | 0.18±0.16 | **0.28±0.12** | 0.80 |
|  |  | original | 107 | 0.60±0.24 | **0.28±0.16** | 0.24±0.30 | 0.57 |
|  |  | selected k best | 40 | 0.58±0.17 | 0.16±0.14 | 0.19±0.11 | 0.88 |
|  | Combined | all | 1926 | 0.64±0.19 | **0.23±0.20** | 0.22±0.16 | 0.46 |
|  |  | imaging (original) | 159 | **0.65±0.19** | 0.22±0.20 | **0.23±0.13** | 0.48 |
|  |  | selected k best | 100 | 0.64±0.26 | 0.20±0.21 | 0.22±0.14 | 0.40 |
|  |  | excluding correlated features | 84* | **0.65±0.20** | 0.21±0.19 | 0.20±0.15 | 0.50 |

**Supplementary Figure 1**. Frequency of missing values in the clinical and blood markers dataset of n=558 patients.


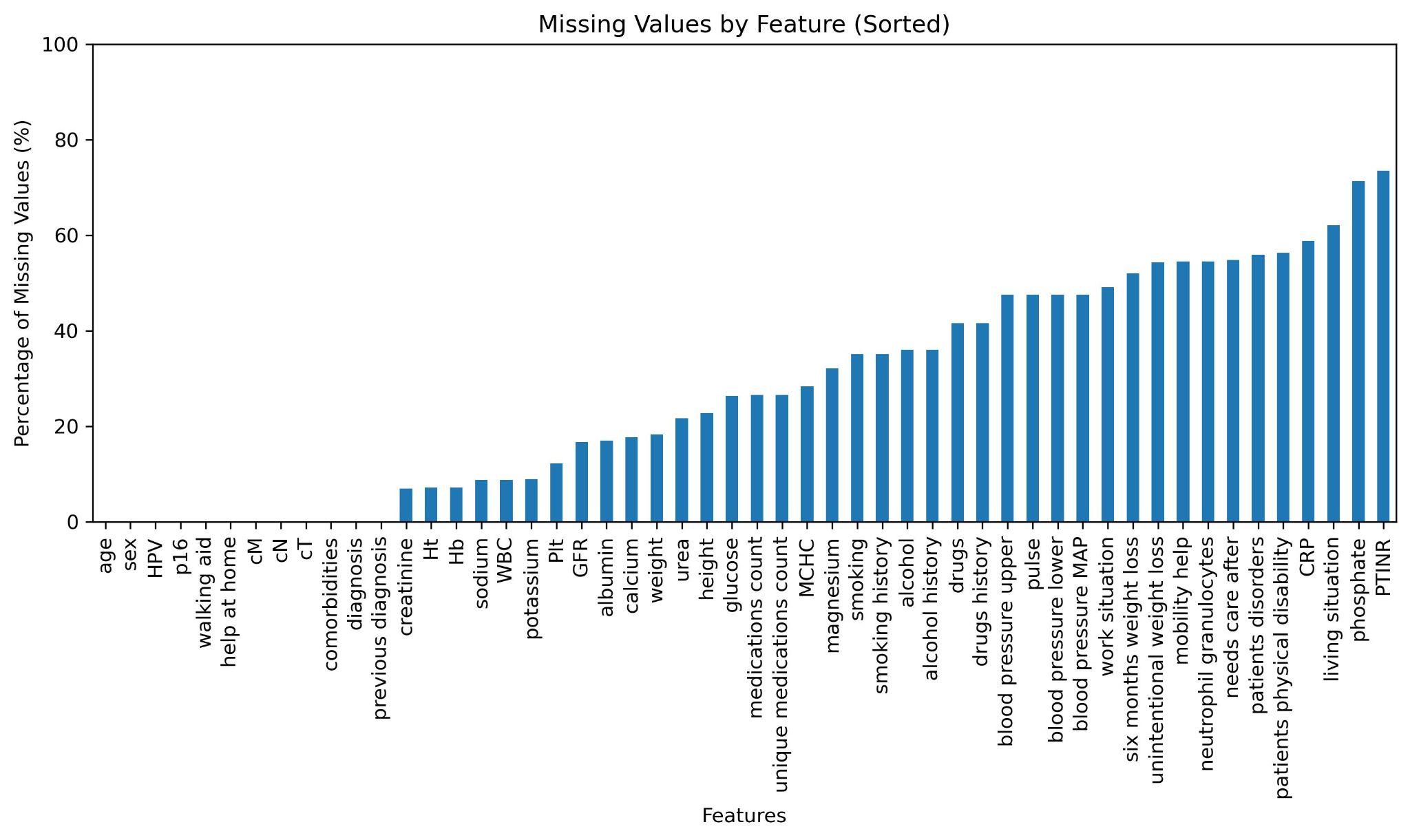

Supplement: Supplementary file 1 — Supplementary file1 (DOCX 230 KB) [file 11548_2025_3539_MOESM1_ESM.docx]
